# Supplementary material for: Intestinal fibroblastic reticular cell niches control innate lymphoid cell homeostasis and function
Source: Nat Commun. 2022 Apr 19;13:2027. doi: 10.1038/s41467-022-29734-2 (PMC9018819; doi:10.1038/s41467-022-29734-2)
Supplement: Supplementary file 1 — Supplementary Information [file 41467_2022_29734_MOESM1_ESM.pdf]

Supplementary information

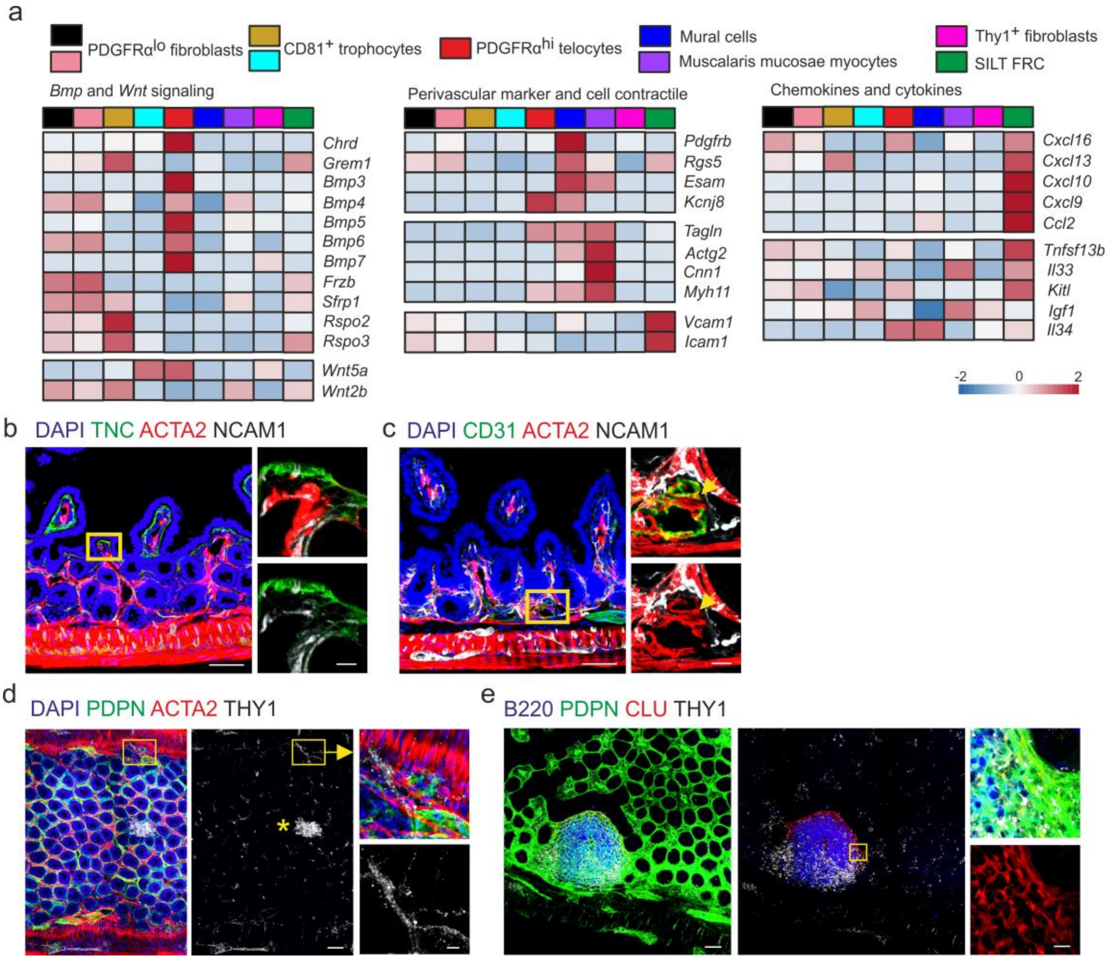

Supplementary Fig. 1. Single cell RNA sequencing analysis and confocal microscopy of lamina propria fibroblasts in the small intestine. a, Heatmap of the scaled gene expression of lamina propria fibroblastic stromal cell markers and curated genes related to *Bmp* and *Wnt* signaling pathways, perivascular markers, cell contractile elements, chemokines and cytokines. **b-e**, Images showing small intestine sections stained with the indicated markers. Scale bar, 50 µm. Boxed areas in (**b-e**) indicate the magnified regions shown in right panels. Scale bar, 10 µm. (**b-e**) Images are representative of at least 6 mice.

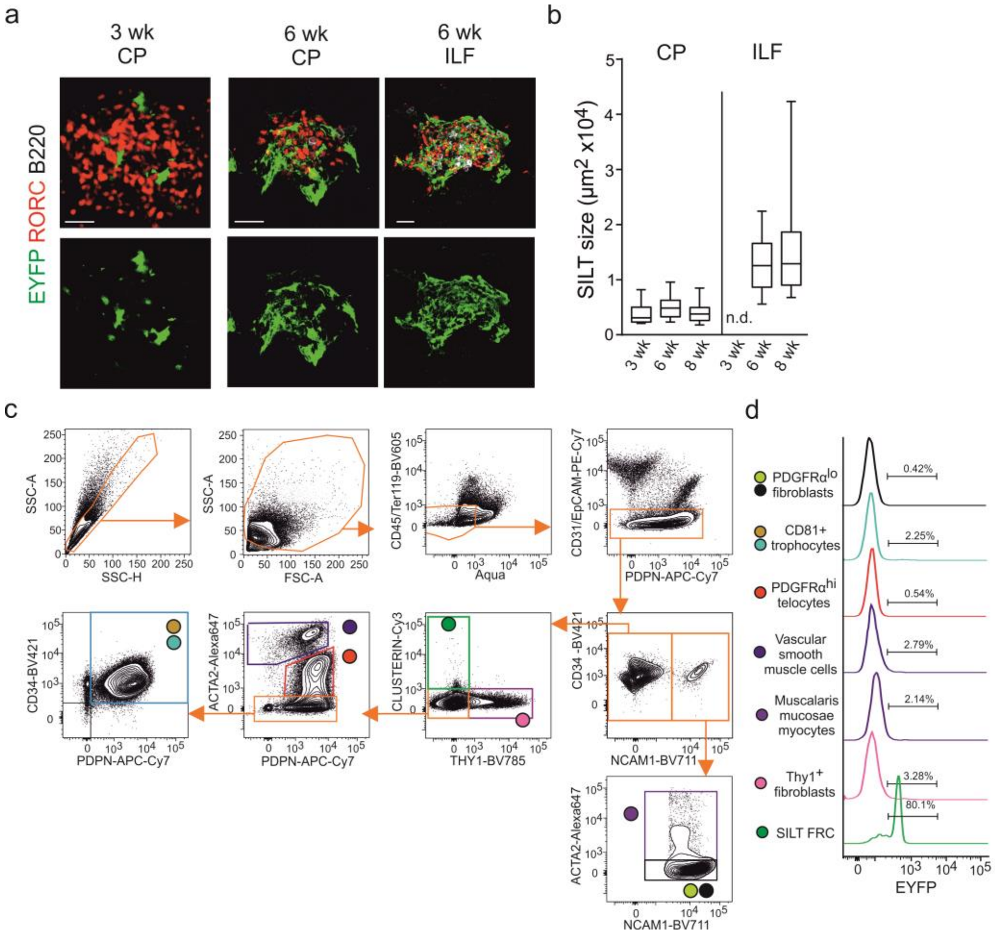

Supplementary Fig. 2. Ccl19-EYFP transgene activity in developing SILT structures. a, Representative confocal microscopy images of SILT structures harvested from 3 or 6 week (wk) old Ccl19-EYFP mice. Scale bar, 30 µm. **b**, Quantification of SILT size from 3 and 6 wk old Ccl19-EYFP mice; n.d., not detectable. Box areas represent median ± interquartile ranges. Whiskers indicate the minima to the maxima of the dataset. **c-d**, Representative gating strategy used for the analysis shown in Figure 1h. (**a**) Images are representative of at least 3 mice. (**b**) n = 18, 16 and 25 in CP, n = n.d., 26, 26 in ILF from 6, 6 and 7 biological replicates in 3 wk, 6 wk and 8 wk old mice respectively from at least 3 independent experiments. (**c-d**) n = 4 mice from 2 independent experiments.

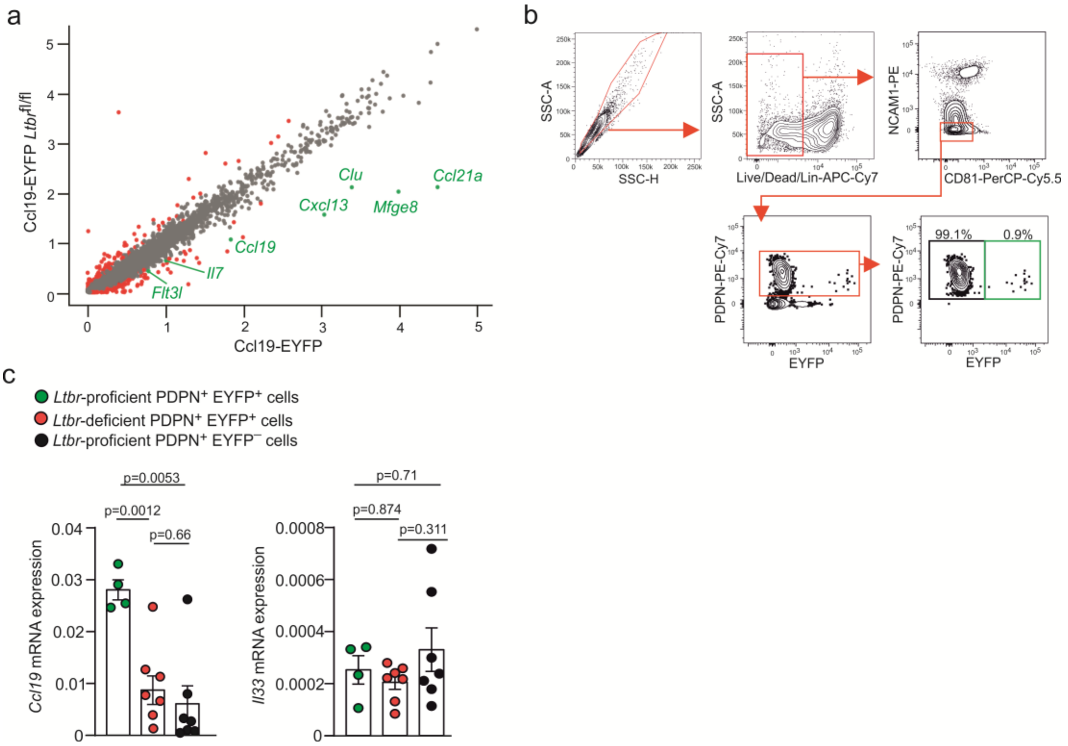

Supplementary Fig. 3. Differential expression of niche factors in *Ltbr*-deficient SILT FRCs. a, Scatter plot of differentially expressed genes in Ccl19-EYFP and Ccl19-EYFP *Ltbr*$^{fl/fl}$ SILT FRC based on scRNA-seq analysis. **b,** Flow cytometry-based sorting strategy to isolate PDPN^{+} EYFP^{+} SILT FRCs and PDPN^{+} EYFP^{-} lamina propria fibroblasts from the small intestine of Ccl19-EYFP and Ccl19-EYFP *Ltbr*$^{fl/fl}$ mice as used in Figure 2f. Lineage (Lin) defined by markers CD45, Ter119, EpCAM and CD31. **c**, Relative expression of *Ccl19* and *Il33* in FACS-sorted PDPN^{+} EYFP^{+} SILT FRCs and PDPN^{+} EYFP^{-} lamina propria fibroblasts from the small intestine of Ccl19-EYFP and Ccl19-EYFP *Ltbr*$^{fl/fl}$ mice as measured by real-time PCR. (**c**) n = 4, 7 and 7 mice respectively. Data represent mean ±SEM. Statistical analyses were performed using a one-way ANOVA.

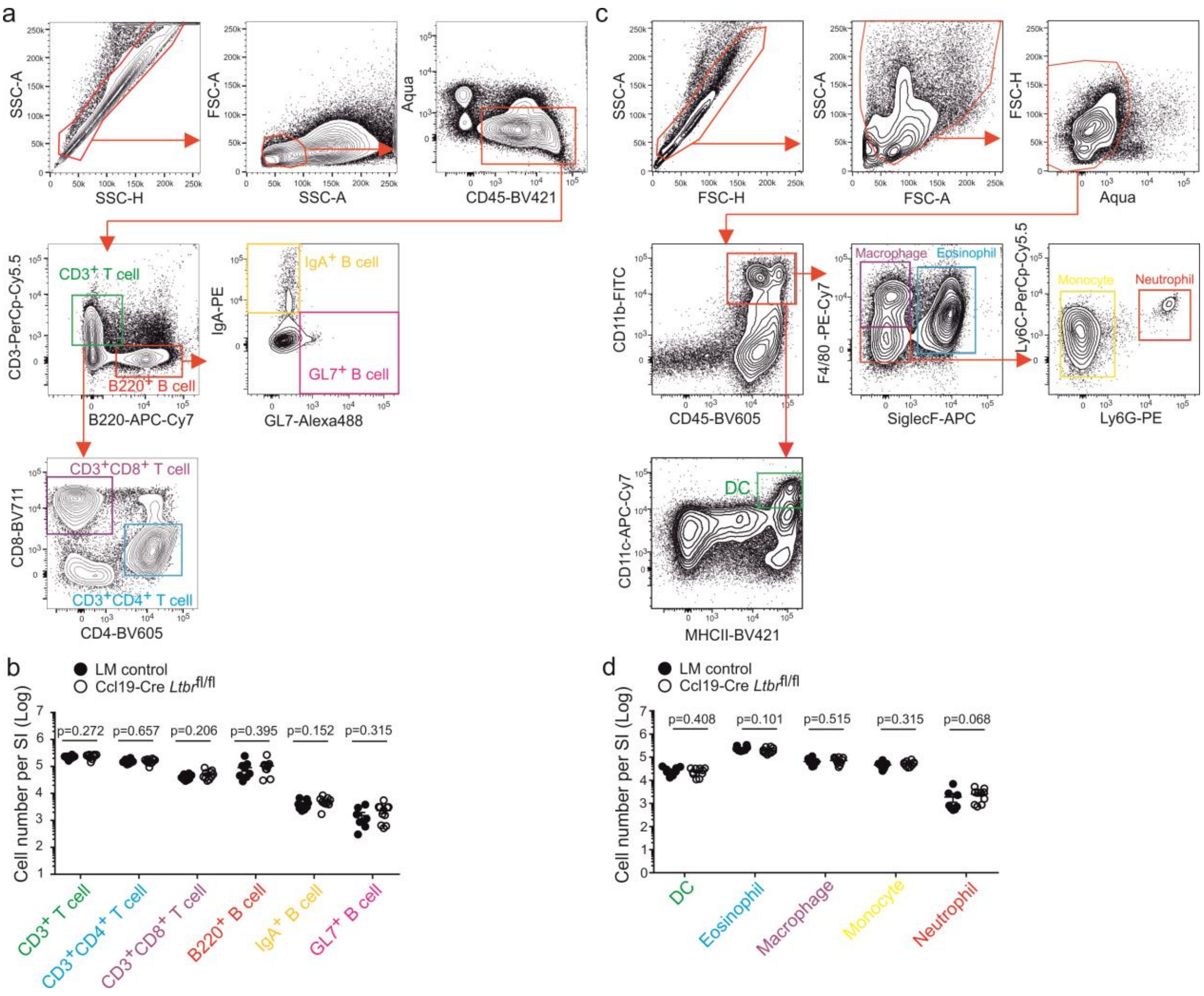

Supplementary Fig. 4. Quantification of lymphocytes and myeloid cells from Ccl19-EYFP *Ltbr*$^{fl/fl}$ mice and co-housed littermate controls. a-d, Flow cytometric analysis to determine the absolute numbers of T cells, B cell and T/B cell subsets (**a, b**), and dendritic cells (DC), eosinophils, macrophages, monocytes and neutrophils (**c, d**) from the small intestine of Ccl19-EYFP *Ltbr*$^{fl/fl}$ mice and co-housed littermate controls (LM control). (**a-d**) n = 10 and 8 mice respectively from 3 independent experiments. Data represent mean ±SEM. Statistical analyses were performed using a non-parametric two-tailed Mann-Whitney test.

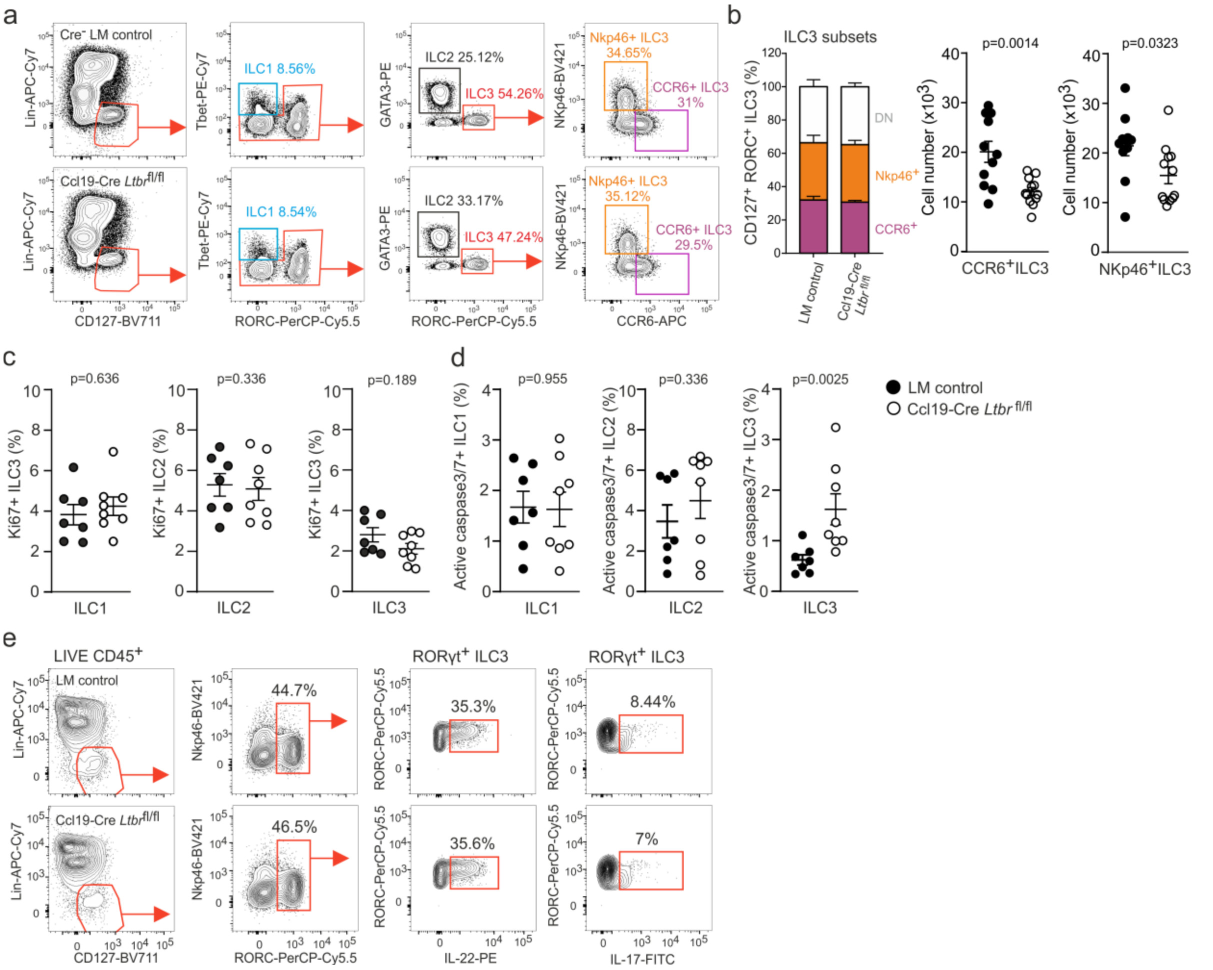

Supplementary Fig. 5. Gating strategy for ILC subset determination in the small intestine of Ccl19-EYFP *Ltbr*$^{fl/fl}$ mice and co-housed littermate controls. a, Representative gating strategy using the markers indicated in Figure 3a-c. Lineage (Lin) defined by markers CD19, CD3, GR1, Ter119 and CD5. **b,** Percentage and absolute cell numbers of CCR6^{+} ILC3 and Nkp46^{+} ILC3. **c-d,** Percentage of Ki67^{+} ILCs and Caspase 3/7^{+} ILCs isolated from Ccl19-EYFP *Ltbr*$^{fl/fl}$ mice and co-housed littermate controls (LM control). **e,** Representative gating strategy using the markers indicated in Figure 3d. (**b**) n = 11 and 12 mice from 4 independent experiments, mean ± SEM. (**c-d**) n = 7 and 8 mice from 3 independent experiments, mean ± SEM. Statistical analyses were performed using the unpaired two-tailed Student's t test (**b**) and the non-parametric two-tailed Mann-Whitney test (**c, d**).

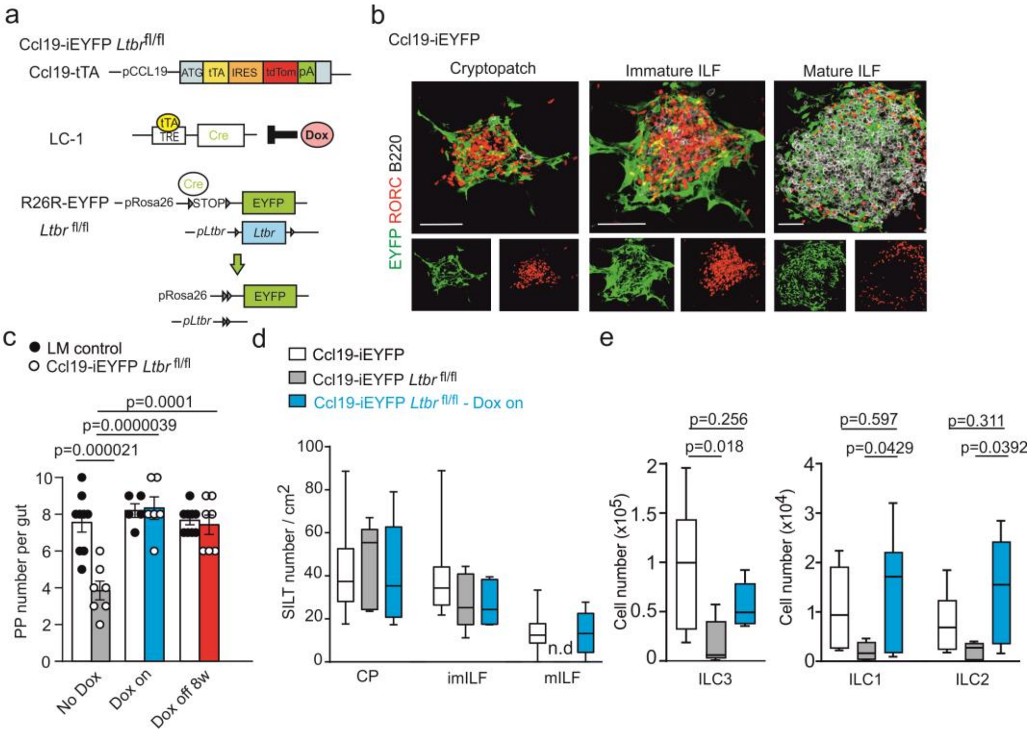

Supplementary Fig. 6. The formation of Peyer's patches, SILT structures and ILC numbers in timed ablation of *Ltbr* expression in SILT FRCs. a, Schematic description of the inducible Ccl19-iEYFP *Ltbr*$^{fl/fl}$ model. **b,** Confocal microscopic analysis of EYFP^{+} SILT FRC networks in SILT structures in Ccl19-iEYFP mice. Scale bar, 50 µm. **c,** Peyer's patch numbers in Ccl19-iEYFP *Ltbr*$^{fl/fl}$ mice and co-housed littermate controls (LM control) without Dox treatment (no DOX), under conditions of permanent treatment with Dox starting during the pregnancy of the dam (Dox on), Dox treatment starting during the pregnancy of the dam and Dox withdrawal at the age of 8 weeks (Dox off 8 wk). **d, e,** Number of SILT structures and **(d)** enumeration of ILC subsets **(e)** in the small intestine of Ccl19-iEYFP and Ccl19-EYFP *Ltbr*$^{fl/fl}$ mice under the indicated Dox treatment conditions. (**b**) Images are representative of at least 6 mice. (**c**) n = 9 and 7 mice in no Dox condition, n = 5 and 6 mice in Dox on condition, n = 9 and 7 mice in Dox off 8 wk condition of co-housed littermate controls and Ccl19-iEYFP *Ltbr*$^{fl/fl}$ mice from at least 3 independent experiments. **(d)** n = 11 of Ccl19-iEYFP mice, n = 5 of Ccl19-EYFP *Ltbr*$^{fl/fl}$ mice in no Dox condition, and n = 5 of Ccl19-iEYFP *Ltbr*$^{fl/fl}$ mice in Dox on condition. Box areas represent median ± interquartile ranges. Whiskers indicate the minima to the maxima of the dataset. (**e**) n = 6 of Ccl19-iEYFP mice and n = 5 of Ccl19-iEYFP *Ltbr*$^{fl/fl}$ mice in no Dox condition, and n = 7 of Ccl19-iEYFP *Ltbr*$^{fl/fl}$ mice in Dox on condition. Box areas represent median ± interquartile ranges. Whiskers indicate the minima to the maxima of the dataset. Statistical analyses were performed using one-way ANOVA (**c - e**).

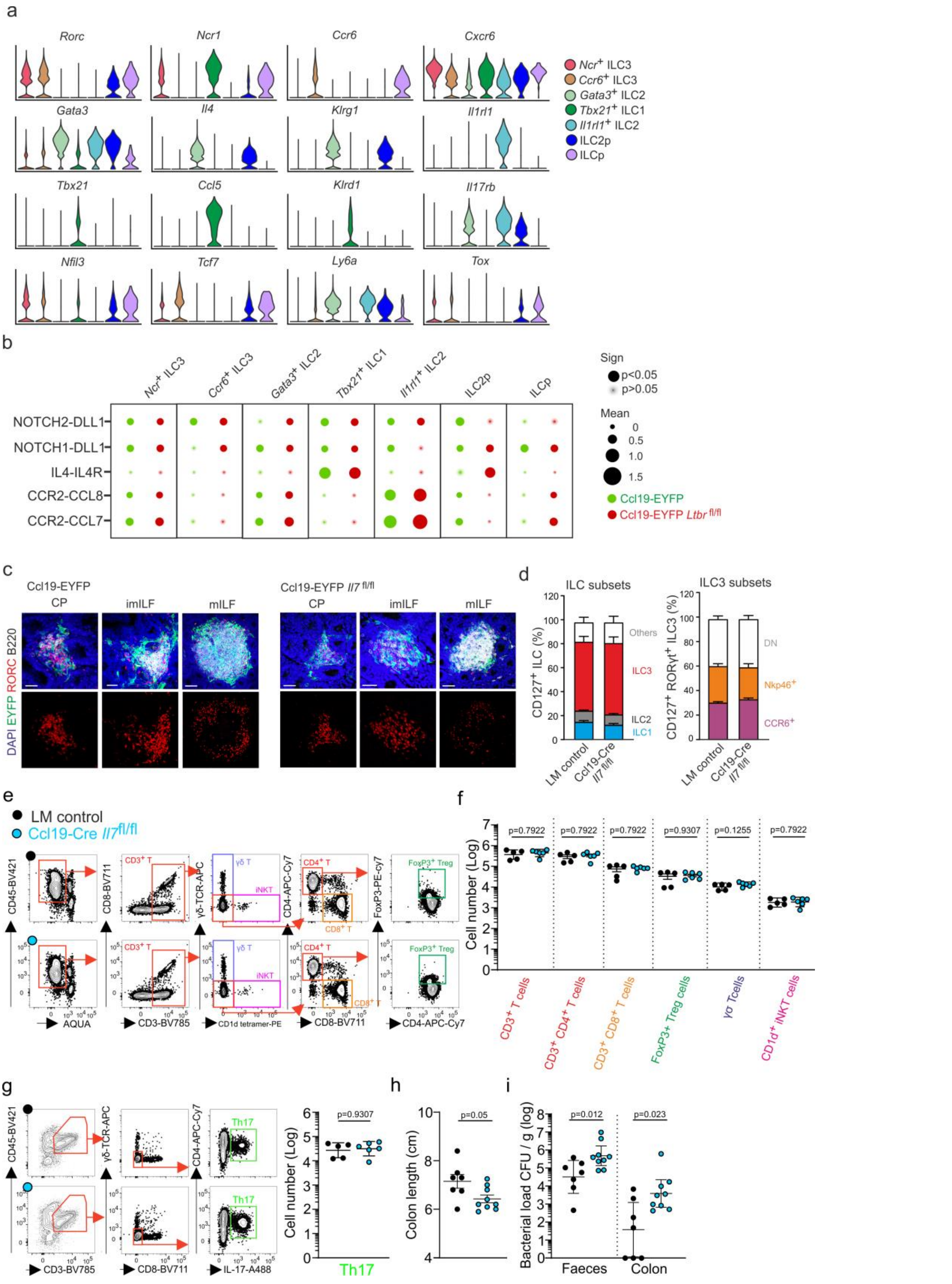

Supplementary Fig. 7. Interactome analysis and in vivo validation of FRC-ILC crosstalk. a, Violin plots depicting subset-specific expression of the indicated genes in ILC subsets. **b,** Predicted intensity of ligand and receptor interaction between SILT FRCs and ILCs from Ccl19-EYFP and Ccl19-EYFP *Ltbr*$^{fl/fl}$ mice based on the CellPhone analysis. The opacity of the circles indicates the degree of statistical signficance and the size of the circles provide a measure

for the interaction intensity. **c,** Representative images of SILT structures in Ccl19-iEYFP and Ccl19-iEYFP *Il7*$^{fl/fl}$ mice analyzed by confocal microscopy after staining with the indicated antibodies. Scale bar, 30 μm in CP and imILF, 50 μm in mILF. Images are representative of 3 mice from each genotype. **d,** ILC and ILC3 subset composition in the lamina propria of Ccl19-Cre *Il7*$^{fl/fl}$ and co-housed littermate controls. n = 7 and 7 mice from 3 independent experiments from Ccl19-Cre *Il7*$^{fl/fl}$ and co-housed littermate controls (LM control). Data represents mean ± SEM. **e,** Representative gating strategy of T cell subsets with flow cytometric analysis from Ccl19-Cre *Il7*$^{fl/fl}$ and co-housed littermate controls. **f,** Cell numbers of different T cell subsets based on the gating in (**e**). **g,** Representative gating of Th17 cells and Th17 cell numbers from Ccl19-Cre *Il7*$^{fl/fl}$ and co-housed littermate controls. n = 6 and 5 mice from 2 independent experiments from Ccl19-Cre *Il7*$^{fl/fl}$ and co-housed littermate controls. Data represents geometric mean ± SD. (**f-g**) **h-i**, Colon length (**h**) and bacterial concentration in faeces and colonic tissue (**i**) on day 11 after C. *rodentium* infection of Ccl19-Cre *Il7*$^{fl/fl}$ mice and co-housed littermate controls. (**h-i**) n = 9 and 7 mice from 2 independent experiments from Ccl19-Cre *Il7*$^{fl/fl}$ and co-housed littermate controls. Data represents mean ± SEM in (**h**) and geometric mean ± SD in (**i**). Statistical analyses were performed using the non-parametric Mann-Whitney test (**f - i**).
