# Supplementary material for: Intestinal fibroblastic reticular cell niches control innate lymphoid cell homeostasis and function
Source: Nat Commun. 2022 Apr 19;13:2027. doi: 10.1038/s41467-022-29734-2 (PMC9018819; doi:10.1038/s41467-022-29734-2)
Supplement: Supplementary file 3 — Description of Additional Supplementary Files [file 41467_2022_29734_MOESM3_ESM.pdf]

Description of Additional Supplementary Files

Supplementary Data 1: Differentially expressed marker genes of SILT FRCs. Marker genes have been determined based on average expression (avg_log2FC) and adjusted p value (p_val_adj) in the SILT FRC cluster compared to all other clusters across the dataset.
