# Supplementary material for: Intestinal fibroblastic reticular cell niches control innate lymphoid cell homeostasis and function
Source: Nat Commun. 2022 Apr 19;13:2027. doi: 10.1038/s41467-022-29734-2 (PMC9018819; doi:10.1038/s41467-022-29734-2)
Supplement: Supplementary file 5 — Reporting Summary [file 41467_2022_29734_MOESM5_ESM.pdf]

nature portfolio

Corresponding author(s): Burkhard Ludewig

Last updated by author(s): 16/2/22

Reporting Summary

Nature Portfolio wishes to improve the reproducibility of the work that we publish. This form provides structure for consistency and transparency in reporting. For further information on Nature Portfolio policies, see our Editorial Policies and the Editorial Policy Checklist.

Statistics

For all statistical analyses, confirm that the following items are present in the figure legend, table legend, main text, or Methods section.

n/a	Confirmed	
☐	☒	The exact sample size (n) for each experimental group/condition, given as a discrete number and unit of measurement
☐	☒	A statement on whether measurements were taken from distinct samples or whether the same sample was measured repeatedly
☐	☒	The statistical test(s) used AND whether they are one- or two-sided<br>*Only common tests should be described solely by name; describe more complex techniques in the Methods section.*
☒	☐	A description of all covariates tested
☐	☒	A description of any assumptions or corrections, such as tests of normality and adjustment for multiple comparisons
☐	☒	A full description of the statistical parameters including central tendency (e.g. means) or other basic estimates (e.g. regression coefficient) AND variation (e.g. standard deviation) or associated estimates of uncertainty (e.g. confidence intervals)
☐	☒	For null hypothesis testing, the test statistic (e.g. F, t, r) with confidence intervals, effect sizes, degrees of freedom and P value noted<br>*Give P values as exact values whenever suitable.*
☒	☐	For Bayesian analysis, information on the choice of priors and Markov chain Monte Carlo settings
☒	☐	For hierarchical and complex designs, identification of the appropriate level for tests and full reporting of outcomes
☒	☐	Estimates of effect sizes (e.g. Cohen's d, Pearson's r), indicating how they were calculated

Our web collection on statistics for biologists contains articles on many of the points above.

Software and code

Policy information about availability of computer code

Data collection	FACSDiva (BD Biosciences, v8.0.1) , FACSChorus (BD Biosciences, v1.3), ZEN (Zeiss, v14.0.18.201),The QuantStudioTM Design & Analysis Software v1.5.1
Data analysis	FlowJo (Treestar Inc., v10), Prism (Graphpad v7), R (v.3.6.2), CellRanger (v3.0.2), scater R/Bioconductor package (v1.14.6), Seurat R package (v.3.1.4), pheatmap R package (v.1.0.12), CellPhone-DB (v.2.1.7), python v.3.7.0, Imaris (v7 and v8)

For manuscripts utilizing custom algorithms or software that are central to the research but not yet described in published literature, software must be made available to editors and reviewers. We strongly encourage code deposition in a community repository (e.g. GitHub). See the Nature Portfolio guidelines for submitting code & software for further information.

Data

Policy information about availability of data

All manuscripts must include a data availability statement. This statement should provide the following information, where applicable:

- Accession codes, unique identifiers, or web links for publicly available datasets
- A description of any restrictions on data availability
- For clinical datasets or third party data, please ensure that the statement adheres to our policy

The scRNA-seq data generated in this study have been deposited in the arrayexpress database (www.ebi.ac.uk/arrayexpress) under accession code E-MTAB-10638 (https://www.ebi.ac.uk/arrayexpress/experiments/E-MTAB-10638/) and E-MTAB-10645 (https://www.ebi.ac.uk/arrayexpress/experiments/E-MTAB-10645/)

Field-specific reporting

Please select the one below that is the best fit for your research. If you are not sure, read the appropriate sections before making your selection.

☒ Life sciences ☐ Behavioural & social sciences ☐ Ecological, evolutionary & environmental sciences

For a reference copy of the document with all sections, see nature.com/documents/nr-reporting-summary-flat.pdf

Life sciences study design

All studies must disclose on these points even when the disclosure is negative.

Sample size	No sample-size calculation was performed. Sample sizes were determined to be adequate based on the reproducibility between independent experiments.
Data exclusions	No data points were excluded
Replication	Experiments were repeated at least two times with sufficient animals per group to demonstrate statistical significance. All experiments were reliably reproduced.
Randomization	Animals were randomly allocated for examination under naive or infection conditions
Blinding	Blinding was not performed. Data analysis was strictly quantitative, with objective outcomes therefore blinding was not relevant for this study.

Reporting for specific materials, systems and methods

We require information from authors about some types of materials, experimental systems and methods used in many studies. Here, indicate whether each material, system or method listed is relevant to your study. If you are not sure if a list item applies to your research, read the appropriate section before selecting a response.

Materials & experimental systems

n/a	Involved in the study	
☐	☒	Antibodies
☒	☐	Eukaryotic cell lines
☒	☐	Palaeontology and archaeology
☐	☒	Animals and other organisms
☒	☐	Human research participants
☒	☐	Clinical data
☒	☐	Dual use research of concern

Methods

n/a	Involved in the study	
☒	☐	ChIP-seq
☐	☒	Flow cytometry
☒	☐	MRI-based neuroimaging

Antibodies

Antibodies used

Antibodies used in this study are listed below (name, clone, supplier name, catalog number, lot number and dilution)

Fixable viability dye eFluor780 eBioscience Cat# 65-0865-14 (Lot:2290915) (1:1000)
Fixable Viability Stain 510 BD Bioscience Cat# 564406 (Lot:0202783) (1:1000)
Anti-Mouse CD3ε Biotin (Clone:145-2C11) BioLegend Cat# 100303; RRID:AB_312668 (Lot:B156785) (1:100)
Anti-Mouse GR1 Biotin(Clone:RB6-8C5) BioLegend Cat# 108403; RRID:AB_313368 (Lot:B268540)(1:100)
Anti-Mouse CD19 Biotin(Clone: 6D5) BioLegend Cat# 115503; RRID:AB_313638 (Lot:B244881) (1:100)
Anti-Mouse Ter119 Biotin(Clone: Ter119) BioLegend Cat# 116203; RRID:AB_313704 (Lot:B256623)(1:100)
Anti-Mouse CD5 Biotin(Clone:53-7.3) BioLegend Cat# 100603; RRID:AB_312732 (Lot:B254316)(1:100)
Anti-Mouse CD127/IL7R BV711(Clone:A7R34) BioLegend Cat# 135035; RRID:AB_2564577 (Lot:B267736) (1:50)
Anti-Mouse CD45.2 BV605(Clone:104) BioLegend Cat# 109841; RRID:AB_2563485(Lot:B310111) (1:100)
Anti-Mouse CD335/Nkp46 BV421(Clone:29A1.4) BioLegend Cat# 137612;RRID:AB_2563104 (Lot:B283369)(1:100)
Anti-Mouse CD196(CCR6) AlexaFluor647 (Clone:140706) BD Bioscience Cat# 557976 (Lot:557976) (1:50)
Anti-Mouse GATA-3 PE (Clone:TWAJ) eBioscience Cat# 12-9966-42 (Lot:4288823)(1:100)
Anti-Mouse RORrt PerCP-Cy5.5 (Clone:Q31-378) BD Bioscience Cat# 562683 (Lot:9053592)(1:50)
Anti-Mouse T-bet PE-Cy7 (Clone:4B10) BioLegend Cat# 644823; RRID:AB_2561760 (Lot:4341621) (1:100)
Anti-Mouse EOMES AlexaFluor488 (Clone:Dan11mag) eBioscience Cat# 53-4875-82(Lot:1994042) (1:100)
Anti-Mouse B220/CD45R APC-Cy7 (Clone:RA3-6B2) eBioscience Cat# 552094(Lot:B266028) (1:200)
Anti-Mouse CD8 BV711(Clone:53-6.7) BioLegend Cat# 100747; RRID:AB_11219594 (Lot:B283373) (1:100)
Anti-Mouse CD4 BV605(Clone:RM4-5) BioLegend Cat# 100547;RRID:AB_11125962 (Lot:B339102) (1:100)
Anti-Mouse CD45.2 BV421(Clone:104) BioLegend Cat# 109831;RRID:AB_10900256(Lot:B310543) (1:100)

Anti-Mouse CD3ε PerCP(Clone:145-2C11) BioLegend Cat# 100325;RRID:AB_893319 (Lot:B266044) (1:100)
Anti-Mouse/Human GL-7 AlexaFluor488 (Clone:GL-7) BioLegend Cat# 144611; RRID:AB_2563284 (Lot:B256896)(1:100)
Anti-Mouse IgA PE(Clone:11-44-2) eBioscience Cat# 12-5994-81(Lot:2090626) (1:20)
Anti-Mouse CD11c APC-Cy7 (Clone:N418) BioLegend Cat# 117323;RRID:AB_830646 (Lot:B237079) (1:100)
Anti-Mouse I-A/I-E BV421(Clone:M5/114.15.2) BioLegend Cat# 107631;RRID:AB_10900075 (Lot:B285303) (1:100)
Anti-Mouse Siglec-F APC(Clone:ES22-10D8) Miltenyi Biotec Cat# 130-123-816 (Lot:5190307128)(1:100)
Anti-Mouse Ly6C PerCP-Cy5.5 (Clone:AL-21) BD Bioscience Cat# 560525 (Lot:560525) (1:100)
Anti-Mouse/Human CD11b AlexaFluor488(Clone:M1/70) BioLegend Cat# 101219; RRID:AB_493545 (Lot:101206) (1:100)
Anti-MouseLy6G PE(Clone:1A8) BioLegend Cat# 127607; RRID:AB_1186104 (Lot:551461)(1:100)
Anti-Mouse F4/80 PE-Cy7(Clone:BM8) BioLegend Cat# 123113;RRID:AB_893490 (Lot:B237342) (1:100)
Anti-Mouse/Human Ki67 AlexaFluor488(Clone:11F6) BioLegend Cat# 151204;RRID:AB_2566800 (Lot:B295927)(1:200)
Anti-Mouse IL17A AlexaFluor488(Clone:TC11-18H10.1) BioLegend Cat# 506909;RRID:AB_536011 (Lot:B259047) (1:50)
Anti-Mouse IL22 PE(Clone:1H8PWSR) eBioscience Cat# 12-7221-82 (Lot:4317946) (1:20)
Anti-Mouse CD81 PerCP-Cy5.5 (Clone:Eat-2) BioLegend Cat# 104911; RRID:AB_2562994(Lot:B295898) (1:100)
Anti-Mouse PDPN APC-Cy7 (Clone:8.1.1) BioLegend Cat# 127417; RRID:AB_2629803(Lot:B236380) (1:200)
Anti-Mouse/Human Alpha-Smooth Muscle Actin eFluor660 (Clone:1A4) eBioscience Cat# 50-9760-82 (Lot:2060395) (1:100)
Anti-Mouse CD90.2 BV785 (Clone:30-H12) BioLegend Cat# 105331; RRID:AB_2562900 (Lot:B289707)(1:100)
Anti-Mouse CD56/NCAM-1 BV711 (Clone:809220) BD Bioscience Cat# 748099 (Lot:9212892) (1:100)
Anti-Mouse TER-119/Erythroid Cells BV605 (Clone:TER-119) BioLegend Cat# 116239; RRID:AB_2562447 (Lot:B277986) (1:100)
Anti-Mouse CD34 BV421 (Clone:MEC14.7) BioLegend Cat# 119321; RRID:AB_10900980 (Lot:B263623)(1:100)
Anti-Mouse CD31 PE-Cy7 (Clone:390) BioLegend Cat# 102418; RRID:AB_830757 (Lot:B264590) (1:100)
Anti-Mouse CD326 (Ep-CAM) PE-Cy7 (Clone:G8.8) BioLegend Cat# 118216; RRID:AB_1236471 (Lot:B282357)(1:100)
Anti-Mouse TCR γ/δ APC (Clone:GL3) BioLegend Cat# 118115; RRID:AB_1731824(Lot:B266832) (1:100)
Anti-Mouse FOXP3 PE-Cy7 (Clone: FJK-16s) eBioscience Cat# 25-5773-82 (Lot:E07638-1632)(1:100)
Goat Anti-Mouse Clusterin (Clone:AF2747) R&D system Cat# AF2747 (Lot:VHY0213121)(1:200)
Anti-Mouse/Human CD45R/B220 AlexaFluor647 (Clone:RA3-6B2) BioLegend Cat# 103226; RRID:AB_389330(Lot:B301693) (1:500)
Syrian Hamster Anti-Mouse Podoplanin (Clone:8.1.1) BioLegend Cat# 127401;RRID:AB_1089186 (Lot:B290022) (1:500)
Goat Anti-Mouse/Human NCAM-1/CD56 (Clone:AF2408) R&D system Cat# AF2408 (Lot:VOK0219091) (1:500)
Anti-Mouse/Human Alpha-Smooth Muscle Actin Cy3 (Clone:1A4) Merck Cat# C6198 (Lot:0000099391)(1:500)
Rabbit Anti-Full-Length GFP Polyclonal Antibody (Clone:AF2408) Takara Bio Clontech Cat# 632592 (Lot:2007065)(1:500)
Biotin Anti-Mouse CXCL13/BLC/BCA-1 (Clone:BAF470) R&D system Cat# BAF470 (Lot:DAD0314121)(1:100)
Biotin Anti-Human/Mouse TenascinC (Clone:MAB2138) R&D system Cat# MAB2138 (Lot:KLC0719101)(1:500)
Anti-Mouse CD31 AlexaFluor647 (Clone:MEC13.3) BioLegend Cat# 102515; RRID:AB_2161030 (Lot:B308659)(1:500)
Biotin Anti-Mouse CD90.2 (Thy1.2) (Clone:53-2.1) BioLegend Cat# 140314; RRID:AB_10643274(Lot:B110532) (1:500)
Anti-Mouse CD54 BV421 (Clone:3E2) BD Bioscience Cat# 565987 (Lot:9058539)(1:100)
Rat Anti-Mouse ROR gamma t (Clone:AFKJS-9) eBioscience Cat# 14-6988-82(Lot:2269906) (1:50)
Alexa488-conjugated anti-rabbit-IgG Jackson Immunotools Cat# 711-546-152 (Lot:146648) (1:500)
Alexa488-conjugated anti-rat-IgG Jackson Immunotools Cat# 112-545-003(Lot:132978) (1:500)
Dylight549-conjugated anti-syrian hamster-IgG Jackson Immunotools Cat# 107-505-142 (Lot:84476)(1:500)
Cy3-conjugated anti-Biotin Jackson Immunotools Cat# 200-162-211 (Lot:154103) (1:500)
Alexa647-conjugated anti-Biotin Jackson Immunotools Cat# 200-602-211 (Lot:146233)(1:500)
Alexa647-conjugated anti-goat-IgG Jackson Immunotools Cat# 705-605-003 (Lot:147708)(1:500)
Cy3-conjugated anti-goat-IgG Jackson Immunotools Cat# 705-605-003(Lot:156363) (1:500)
Biotin-conjugated anti-rat-IgG Jackson Immunotools Cat# 112-065-003 (Lot:100267)(1:500)

Validation

All antibodies came from commercial vendors, and were validated by the manufacturers on their official website. Antibodies were evaluated with positive signals through comparing the signals to cognate antibody isotype control.

Animals and other organisms

Policy information about studies involving animals; ARRIVE guidelines recommended for reporting animal research

Laboratory animals

All mouse strains were on a C57BL/6NCrl genetic background and maintained in individually ventilated cages under specific pathogen-free conditions. C57BL/6N-Tg(Ccl19-Cre)489Biat (Ccl19-Cre), C57BL/6N-Tg(Ccl19-tTA)688BIAT (Ccl19-tTA) and Ltbrtm1.1Thhe (LTβRfl/fl) strains were described previously. C57BL/6N-(IL7fl/fl)tm1Iku (IL7fl/fl) are kindly provided by Koichi Ikuta (Kyoto University, Japan). B6.129X1-Gt(ROSA)26Sortm1(EYFP)Cos/J (R26R-EYFP) mice were purchased from The Jackson Laboratories and the LC1 strain66 was kindly provided by Dr. Fendler (Max Delbrück in Center of Molecular Medicine, Berlin, Germany). Non-antibiotic 4-Epidoxycycline (Dox) was kindly provided by Dr. Rodewald in University of Leipzig, Germany. 4-Epidoxycycline was administered to pregnant dams in the drinking water (50 µg/ml) and maintained after weaning. The application of 4-Epidoxycycline was withdrawal after the age of 8 weeks as the time scheme shown in Figure 4a. All experiments were performed with 8 to 12 weeks old mice, except for Figure 4 and Supplementary Figure 2a-b and 6c-e. Experiments in Supplementary Figure 2a-b were performed with age 3, 6 and 8 week-old mice as indicated in the figure legend. Experiments in Figure 4 and Supplementary Figure 6c-e were performed with 8, 10 and 16 week-old mice as indicated in Figure 4a and figure legend. The sex ratio of female and male is approximately 1:1. All mice were housed in the Institute of Immunobiology, Kantonsspital St. Gallen under specific-pathogen-free (SPF) conditions at 22°C±2 and 50±5% humidity in a 12/12-h light/dark cycle and provided ad libitum access to food and water.

Wild animals

None

Field-collected samples

None

Ethics oversight	Experiments were performed in accordance with Swiss federal and cantonal guidelines (Tierschutzgesetz) under the permission numbers SG01/18, SG08/18, SG04/20, SG01/18 and SG24/2020 granted by the Veterinary Office of the Canton of St. Gallen.

Note that full information on the approval of the study protocol must also be provided in the manuscript.

Flow Cytometry

Plots

Confirm that:

- [x] The axis labels state the marker and fluorochrome used (e.g. CD4-FITC).
- [x] The axis scales are clearly visible. Include numbers along axes only for bottom left plot of group (a 'group' is an analysis of identical markers).
- [x] All plots are contour plots with outliers or pseudocolor plots.
- [x] A numerical value for number of cells or percentage (with statistics) is provided.

Methodology

Sample preparation	A description of the sample preparation for the flow cytometry is detailed in the methods section
Instrument	LSR Fortessa 2 BD Biosciences, FACS Melody BD Biosciences
Software	FACSDiva was used to collect the data and FlowJO v10 (Treestar inc.) to analyze the data. For cell sorting for scRNA-Seq, FACSChorus was used to set up cell sorting, and R Studio was used to analyze the transcriptomic data.
Cell population abundance	A test sample was prepared for testing the purity of sorted cells. The purity of the post-sort fraction was determined by flow cytometry using the LSR Fortessa 2.
Gating strategy	For all flow cytometric analysis, cells were first gated on FSC/SSC to exclude cell debris following by FSC-A/FSC-H to exclude doublets. Dead cells were excluded from analysis by gating on viability dye negative staining. Gating strategy for identifying stromal cell population and innate lymphoid cells analyzed in this study is exemplifying in the Extended data figures.

- [x] Tick this box to confirm that a figure exemplifying the gating strategy is provided in the Supplementary Information.
